# Dimensional Accuracy and Measurement Variability in CNC-Turned Parts Using Digital Vernier Calipers and Coordinate Measuring Machines Across Five Materials

**DOI:** 10.3390/ma18122728

**Published:** 2025-06-10

**Authors:** Mohammad S. Alsoufi, Saleh A. Bawazeer, Mohammed W. Alhazmi, Hasan H. Hijji, Hani Alhazmi, Hazzaa F. Alqurashi

**Affiliations:** Department of Mechanical Engineering, College of Engineering and Architecture, Umm Al-Qura University, Makkah 21955, Saudi Arabia; mssoufi@uqu.edu.sa (M.S.A.); mwhazmi@uqu.edu.sa (M.W.A.); hhhijji@uqu.edu.sa (H.H.H.); hahazmi@uqu.edu.sa (H.A.); hfqurashi@uqu.edu.sa (H.F.A.)

**Keywords:** CNC machining, coordinate measuring machine, digital vernier caliper, dimensional metrology, measurement uncertainty, relative error

## Abstract

Attaining dimensional accuracy in CNC-machined parts is essential for high-precision manufacturing, especially when working with materials that exhibit varying mechanical and thermal characteristics. This research provides a thorough experimental comparison of manual and automated metrological systems, specifically the Digital Vernier Caliper (DVC) and Coordinate Measuring Machine (CMM), as applied to five different engineering alloys through five progressively machined axial zones. The study assesses absolute error, relative error, standard deviation, and measurement repeatability, factoring in material hardness, thermal conductivity, and surface changes due to machining. The results indicate that DVC performance is significantly affected by operator input and surface irregularities, with standard deviations reaching 0.03333 mm for Bronze C51000 and relative errors surpassing 1.02% in the initial zones. Although DVC occasionally showed lower absolute errors (e.g., 0.206 mm for Aluminum 6061), these advantages were countered by greater uncertainty and poor repeatability. In comparison, CMM demonstrated enhanced precision and consistency across all materials, with standard deviations below 0.0035 mm and relative errors being neatly within the 0.005–0.015% range, even with challenging alloys like Stainless Steel 304. Furthermore, a Principal Component Analysis (PCA) was conducted to identify underlying measurement–property relationships. The PCA highlighted clear groupings based on sensitivity to error in manual versus automated methods, facilitating predictive classification of materials according to their metrological reliability. The introduction of multivariate modeling also establishes a new framework for intelligent metrology selection based on material characteristics and machining responses. These results advocate for using CMM in applications requiring precise tolerances in the aerospace, biomedical, and high-end tooling sectors, while suggesting that DVC can serve as an auxiliary tool for less critical evaluations. This study provides practical recommendations for aligning measurement techniques with Industry 4.0’s needs for accuracy, reliability, and data-driven quality assurance.

## 1. Introduction

In modern precision manufacturing, dimensional accuracy and surface integrity are not merely technical requirements but strategic enablers of product performance, functional reliability, and lifecycle sustainability [1,2]. These considerations are especially critical in high-value sectors such as aerospace, biomedical devices, and advanced automotive systems, where tight geometric tolerances govern fatigue resistance, regulatory compliance, and functional interoperability [3,4,5,6]. With the emergence of Industry 4.0, the role of dimensional metrology has evolved into a cornerstone for intelligent process controls, digital-twin implementations, and closed-loop quality assurance systems [7].

While high-resolution automated systems continue to gain prominence, manual metrological instruments like the digital vernier caliper (DVC) remain prevalent due to their affordability and ease of use. However, DVC measurements are inherently vulnerable to operator-induced uncertainties, such as angular misalignment, variable contact pressure, and parallax error, which are further amplified by machining-induced surface variability [8,9]. These effects are often compounded by material-dependent responses, including localized heat accumulation, residual stress, and tool-induced surface deformation, particularly in CNC-machined parts [10,11].

To mitigate these issues, coordinate measuring machines (CMMs) offer sub-micron precision enabled by automated probing, volumetric calibration, and thermal compensation [12]. Nevertheless, CMMs can also be affected by surface features such as waviness, burrs, and anisotropic microstructures that may alter probe–surface interactions [13]. Recent studies have shown that intrinsic material characteristics, especially hardness, thermal conductivity, and machinability, directly influence measurement accuracy and repeatability [14,15,16]. For instance, Alsoufi and Bawazeer (2025) demonstrated that high thermal conductivity promotes stable surface formation during machining, thereby reducing downstream dimensional variation, while elevated hardness increases tool wear and geometric deviation [17,18].

Despite this progress, most prior studies (e.g., [9,11,14]) consider measurement errors to be primarily instrument-specific and often treat dimensional accuracy as a static outcome, overlooking how machining progression and spatial changes in surface conditions affect metrological performance. Furthermore, comparative studies are often based on single-point measurements or ideal calibration artifacts, which fail to reflect real-world variations in surface quality or tool wear across the machining path.

To address these limitations this study proposes a zone-resolved comparative analysis of DVC and CMM performance across five engineering materials, Aluminum Alloy 6061, Brass C26000, Bronze C51000, Carbon Steel 1020 Annealed, and Stainless Steel 304 Annealed (supplied by SpecLine Arabia Company Ltd., and Al Mokahal Co., Al-Jubail, Saudi Arabia), with diverse thermal and mechanical properties. Dimensional measurements were collected from five axial zones on each workpiece to account for machining-induced changes. The analysis evaluates absolute error, relative error, standard deviation, and expanded uncertainty in accordance with the Guide to the Expression of Uncertainty in Measurement (GUM) framework.

A key innovation in this study is the application of principal component analysis (PCA) to identify multivariate relationships between material properties and measurement stability. This approach reveals material-specific metrological signatures, enabling predictive classification for intelligent inspection planning. Unlike conventional assessments that treat dimensional accuracy as a static outcome, our framework interprets it as a dynamic function of tool, material, and surface evolution, offering practical guidance for metrology selection in Industry 4.0 environments where adaptability and traceability are critical.

## 2. Materials and Methods

### 2.1. Material Selection and Machining Setup

This study used five engineering materials that were carefully chosen to cover a broad range of properties that affect machining and measurement. The selected materials, Aluminum Alloy 6061, Brass C26000, Bronze C51000, Carbon Steel 1020 Annealed, and Stainless Steel 304 Annealed (supplied by SpecLine Arabia Company Ltd., and Al Mokahal Co., Al-Jubail, Saudi Arabia), differ significantly in hardness, thermal conductivity, and machinability. These differences are important because they influence how the material’s surface behaves during turning and how accurately that surface can be measured afterwards. For example, soft and highly conductive materials like aluminum and brass tend to produce smoother surfaces that are easier to measure. In comparison, harder and less conductive materials like carbon steel and stainless steel may lead to rougher surfaces and more variation in measurement. Bronze provides a middle ground between these extremes. These materials were also selected because they are widely used in real industrial applications such as in the automotive sector, the aerospace sector, and general manufacturing. Their thermal conductivity, Brinell hardness, and machinability values are summarized in Table 1. These values reflect standard industrial specifications, verified through material datasheets provided by SpecLine Arabia Company Ltd., and Al Mokahal Co.

Cylindrical workpieces were processed using a high-precision CNC lathe, model Gate-Eclipse ECL-400, featuring a Fagor control system (Gate Machinery International Ltd., Derbyshire, UK). This system was chosen for its excellent structural rigidity and thermal stability during continuous machining operations. The machining conditions were kept at 22 ± 1 °C and 45 ± 5% relative humidity, following the guidelines of ISO 230-3 [19], which outlines methods for assessing the thermal impact on machine tool performance.

Each workpiece, originally measuring 25 mm in diameter and 45 mm in length, was divided into five equal axial zones (F1 to F5). The centers of these zones were located at distances of 5.85 mm, 14.55 mm, 23.25 mm, 31.95 mm, and 40.65 mm from the chuck face. This segmentation facilitated the spatial analysis of surface and dimensional changes in relation to the distance from the chuck, considering factors such as tool wear evolution, thermal loading, and variations in support stiffness.

Machining parameters were standardized for all materials to examine the impact of material properties. Cutting speeds of 30, 60, 90, and 120 m/min were sequentially applied along with feed rates of 0.05, 0.10, 0.15, and 0.20 mm/rev, all while maintaining a constant depth of cut at 0.25 mm and dry cutting conditions. ISO CNMG120408-PM Tungsten carbide [20] inserts with TiAlN coatings were used, being replaced after five passes or when a flank wear of 0.3 mm was reached in accordance with ISO 3685 guidelines [21].

**Figure 1:**
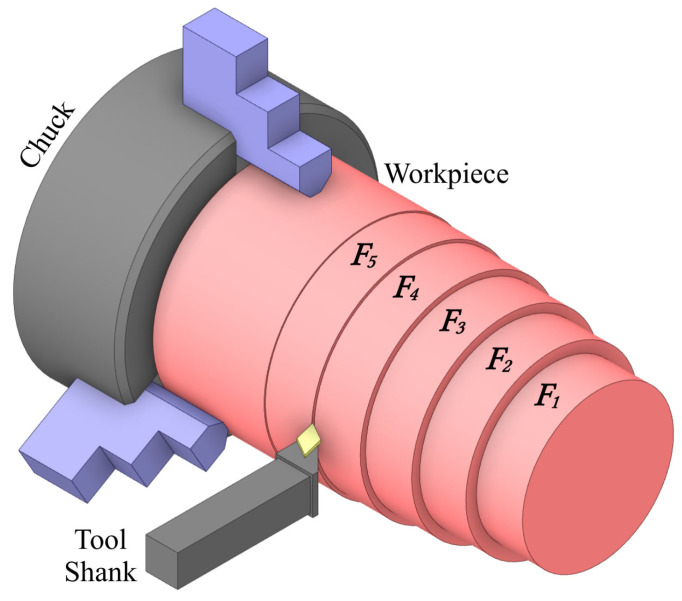
Schematic overview of the experimental setup. The workpiece is divided into five measurement segments (F1–F5), representing distinct axial positions from the free end (F1) toward the chuck (F5).

Workpieces were secured with a precision three-jaw chuck to guarantee dimensional consistency and prevent positional shifts. Tool and spindle alignment were checked prior to each machining cycle, ensuring a setup tolerance within ±0.01 mm in accordance with ISO 230-1 [22]. Figure 1 displays a schematic of the experimental layout, highlighting the machining zones and measurement stations.

### 2.2. Measurement Systems and Calibration Traceability

**Figure 2:**
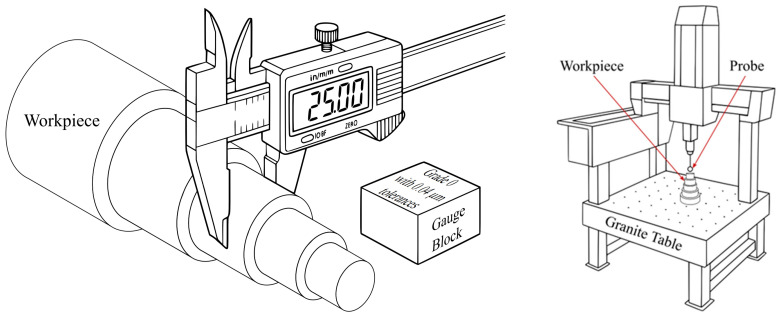
Schematic of the measurement setups: (**left**) digital vernier caliper (DVC) with gauge blocks and workpiece; (**right**) coordinate measuring machine (CMM) with a probe, granite table, and workpiece.

Dimensional evaluations utilized the following two complementary metrological systems: a digital vernier caliper (DVC) for manual measurements and a coordinate measuring machine (CMM) for automated, high-precision assessments. These tools were chosen to illustrate the differing traits of operator-dependent versus computer-controlled dimensional metrology. Figure 2 provides schematic illustrations of both setups, including the DVC with calibration blocks and workpiece and the CMM with a probing head and a granite table in a thermally stabilized environment.

The DVC employed was a Preisser Messtechnik model, boasting a digital resolution of ±0.02 mm and a measurement range of 0–150 mm (Preisser Messtechnik GmbH, Gammertingen, Germany). All measurements took place on a vibration-isolated granite table to reduce external disturbances. Calibration was conducted daily using ISO 3650-certified Grade 0 steel gauge blocks (nominal lengths: 10 mm, 50 mm, and 100 mm) [23], resulting in an average deviation of ±0.015 mm (Mitutoyo Corporation, Kawasaki, Kanagawa, Japan). Calibration results were recorded systematically to ensure traceability in line with international metrology standards.

Manual caliper measurements are subject to Type A uncertainties (random variations from repeatability) and Type B uncertainties (systematic factors like alignment errors, inconsistent pressure, and parallax mistakes). A standardized procedure was implemented to address these issues. The caliper jaws were positioned orthogonally to each cylindrical surface and a torque-limited gripping method was applied to ensure a consistent force. Each feature underwent five measurements, with both the mean and standard deviation calculated to evaluate repeatability. Even with these controls in place, DVC readings were still influenced by surface roughness, elastic recovery, and operator variation, necessitating a thorough uncertainty quantification according to the Guide to the Expression of Uncertainty in Measurement (GUM) framework.

The CMM used was a Millennium DIGI-MET model, rated IP65, offering a volumetric precision of ±0.001 mm across a measurement range of 0–200 mm. This system was manufactured by Millennium Measurement Machines Ltd., Leicestershire, UK, and selected for its robust structural design, precision air-bearing granite base, and superior thermal stability under precision metrological conditions. Calibration was conducted before each session using a 25.000 mm reference sphere and ISO 3650-certified Grade 0 gauge blocks (50 mm and 100 mm) [23]. These procedures conformed to ISO 10360-2 (length measurement error, E_0_, MPE) [24] and ISO 10360-5 (probing form error, PFTU) [25]. All measurements were carried out in a thermally regulated environment at 22 ± 1 °C and 45 ± 5% relative humidity, closely matching the ISO 1-standard reference conditions of 20 ± 0.5 °C and 50 ± 10% RH [26].

CMM measurements were taken with a fully automated probing routine. Each feature was assessed at ten evenly spaced points, utilizing controlled approach angles and probing forces. This automated data collection minimized operator variability and greatly decreased Type A uncertainty. Type B factors such as stylus deflection, thermal expansion of structural elements, and reference artifact deviation were included in a comprehensive expanded uncertainty budget, which was presented at a 95% confidence level (coverage factor *k* = 2).

Both instruments were calibrated using traceable reference standards from national metrology institutes (NMIs), guaranteeing adherence to globally accepted traceability protocols and facilitating a valid comparison between manual and automated measurement results.

### 2.3. Measurement Procedures

Each machined feature underwent five repeated dimensional measurements with both the digital vernier caliper (DVC) and the coordinate measuring machine (CMM). A structured protocol was established to guarantee repeatability, reduce uncertainty, and enable a valid comparison between manual and automated measurement systems.

For DVC measurements the caliper jaws were carefully positioned orthogonally to the cylindrical axis of each feature. A standardized hand pressure technique was used to minimize clamping variability and prevent elastic deformation, especially with softer materials, supported by a mechanical fixture to ensure that the jaws remained parallel when applicable. Significant care was taken to avoid misalignment, angular tilt, or excessive force as each of these could compromise the accuracy of the readings. Each feature underwent five measurements, with full jaw release occurring between repetitions to eliminate any preload bias. The mean and standard deviation of these five measurements were recorded along with continuous monitoring for signs of operator fatigue or systematic drift. The same trained operator conducted all DVC measurements throughout the study to reduce inter-operator variability.

CMM measurements utilized a fully automated probing routine, employing a fixed strategy of ten evenly spaced points for each feature. The angles, speeds, and contact forces of the probing approach were standardized. The probe path was fine-tuned to reduce stylus deflection while ensuring sufficient surface coverage. Temperature compensation and pre-measurement warm-up routines were implemented to guarantee mechanical stability. Three complete scans were performed for each feature to assess local surface variations and confirm machine repeatability. All measurements were conducted on an air-bearing granite table situated in a vibration-isolated, thermally regulated environment.

The environmental conditions were strictly controlled throughout all measurement sessions. The measurement room was maintained at 22 ± 1 °C and 45 ± 5% relative humidity in accordance with ISO 1-standard conditions. All specimens and instruments were allowed to acclimate for at least two hours prior to measurement to ensure thermal uniformity and prevent dimensional changes.

Surface roughness was monitored using contact profilometry on representative machined features. The average surface roughness (*R*_a_) was maintained below 2.5 µm, ensuring minimal contact distortion. For DVC this reduced the influence of roughness on jaw seating, whilst for CMM it preserved the integrity of probe contact and avoided local deflection errors.

By integrating controlled alignment procedures, environmental stabilization, consistent measurements, and surface roughness verification, both manual and automated dimensional assessments were performed under optimized and reproducible conditions. This method allowed for a statistically rigorous comparison of DVC and CMM performance across various materials and machining zones.

### 2.4. Data Analysis Methods

Data gathered from both the digital vernier caliper (DVC) and the coordinate measuring machine (CMM) were carefully analyzed to assess dimensional discrepancies, measurement uncertainty, and repeatability for all machining zones and engineering materials. A systematic analytical method was adopted to facilitate a thorough, statistically sound comparison between the manual and automated measurement systems.

The absolute error ($E_{abs}$) for every feature was determined as the absolute difference between the observed measured value and the nominal target dimension, in accordance with the following expression:

$$E_{abs}=\left|M_{measured}-M_{nominal}\right|,$$

where $M_{measured}$ represents the measured dimension obtained from either the DVC or CMM, while $M_{nominal}$ denotes the nominal value specified in the machining design. To normalize absolute deviations and facilitate comparisons across various feature sizes and materials, the relative error ($E_{rel}$) was calculated using the following:

$$E_{rel}=\left(\frac{E_{abs}}{M_{nominal}}\right)\times 100$$

This relative scaling offered a dimensionless percentage view of measurement deviation, enabling straightforward comparisons across various machining conditions and material types.

Expanded measurement uncertainty (U) was evaluated according to the Guide to the Expression of Uncertainty in Measurement (GUM) methodology [27]. The combined standard uncertainty considered the following three principal contributions: measurement repeatability ($\sigma_{repeatability}$), instrument resolution uncertainty ($\sigma_{resolution}$), and calibration artifact uncertainty ($\sigma_{calibration}$). The expanded uncertainty was calculated using the following relationship:

$$U=k\times \sqrt{{\left(\sigma_{repeatability}\right)}^{2}+{\left(\sigma_{resolution}\right)}^{2}+{\left(\sigma_{calibration}\right)}^{2}}$$

where the value of *k* = 2 was chosen to indicate a 95% confidence level. The measurement was assessed based on deviations seen during the daily calibration verification processes, which utilized ISO 3650-certified gauge blocks and ISO 10360-2 reference spheres, all performed under ISO 1-standard environmental conditions (20 ± 0.5 °C and 50 ± 10% RH).

Alongside uncertainty evaluation, fundamental statistical parameters such as the mean (μ), standard deviation (SD), and coefficient of variation (CV) were computed for each feature to capture the central tendency and variation in the measurements. The coefficient of variation was calculated using the formula:

$$CV=\left(\frac{\sigma_{repeatability}}{\mu }\right)\times 100.$$

This offers a standardized measure of variability that enables the comparison of repeatability among materials, machining zones, and measurement systems, regardless of the nominal dimension scale.

All graphical displays of the measurement results included expanded uncertainty bars that reflect the 95% confidence interval. This approach allowed for a clear depiction of measurement variability and facilitated a thorough evaluation of the precision and stability attributes of both measurement methods under different material properties and machining conditions. A systematic assessment of absolute error, relative error, expanded uncertainty, and measurement repeatability is conducted utilizing the analytic framework, yielding valuable insights into the metrological performance of both manual and automated dimensional measurement systems.

## 3. Results and Discussion

### 3.1. Measurement Accuracy Across Materials: DVC vs. CMM

**Figure 3:**
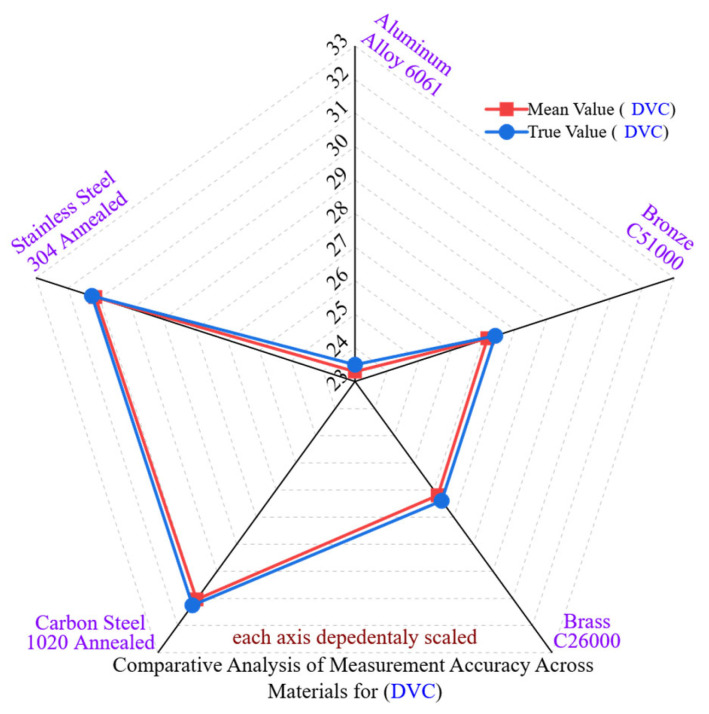
Comparative analysis of measurement accuracy across materials for DVC.

Figure 3 presents a radar analysis comparing dimensional deviations in five engineering materials that were measured with a digital vernier caliper (DVC). The findings indicate that the precision of manual measurements is notably influenced by the properties of the materials, especially hardness and surface condition, due to the DVC’s sensitivity to factors such as operator handling, jaw alignment, and the consistency of tactile contact. Although the DVC is popular for its ease of use, the analysis demonstrates that its accuracy and repeatability can vary significantly among materials with differing machinability and mechanical responses.

Aluminum Alloy 6061 exhibited the highest DVC accuracy among the evaluated materials. The mean deviation from the nominal was −0.2397 mm, accompanied by a standard deviation (SD) of ±0.02974 mm and a coefficient of variation (CV) of 0.1279%. This positive outcome can be linked to aluminum’s low hardness (95 HBW) and high thermal conductivity (167 W/m·K), which facilitate stable machining and minimize surface irregularities that may interfere with manual measurements. Nonetheless, despite the advantageous surface conditions, the CV reveals moderate repeatability limitations due to the instrument’s manual nature.

Brass C26000 exhibited a deviation of −0.2398 mm, with a standard deviation of ±0.02912 mm and a coefficient of variation (CV) of 0.1071%. It achieves a machinability rating of 100% paired with low hardness (70 HBW), which facilitate smooth chip removal and result in fairly uniform surfaces. However, the CV’s closeness to that of aluminum indicates that tactile sensitivity and angularity issues during measurements significantly impact DVC variability, surpassing the influence of material response alone.

Bronze C51000, which is harder (110 HBW) and less machinable (50%), showed a greater deviation of −0.2824 mm, with a standard deviation of ±0.03333 mm and a coefficient of variation of 0.1226%. These outcomes were likely due to increased elastic recovery, localized residual stresses, and the formation of burrs. Moreover, the DVC’s limited ability to adjust to microstructural surface gradients and its reliance on uniform contact pressure diminish its precision in these situations.

Carbon Steel 1020 Annealed, known for its decent machinability rating of 65% and moderate hardness (126 HBW), showed a deviation of −0.2327 mm, with a standard deviation of ±0.01763 mm and a coefficient of variation of 0.0569%. This represented the least variability among the five materials tested, indicating that the material’s machining properties resulted in geometrically consistent features, thereby reducing the effects of operator-induced errors during DVC readings.

Stainless Steel 304 Annealed, known for its highest hardness (190 HBW) and lowest thermal conductivity (16 W/m·K), exhibited a deviation of −0.1312 mm, a standard deviation of ±0.02793 mm, and a coefficient of variation of 0.0893%. Although the mean deviation is smaller, repeatability was still adversely affected by work hardening effects and microstructural variability, which disrupt local surface compliance during jaw contact.

Statistically, the CV values for DVC measurements varied between 0.0569% and 0.1279%. This shows notable variability that weakly correlates with mean deviation but strongly relates to material hardness and surface condition. Consequently, this highlights that absolute accuracy alone is not a dependable indicator of metrological performance in manual systems. Rather, CV and SD offer a more comprehensive understanding of measurement stability.

These findings confirm that DVC, although useful for rapid geometric verification, lacks consistent repeatability when dealing with harder materials, those with lower machinability, or work-hardened surfaces. Consequently, both material characteristics and human elements affect measurement quality. DVC should only be used for initial evaluations or additional checks in high-precision situations, while automated systems are recommended for dependable and traceable dimensional verification.

**Figure 4:**
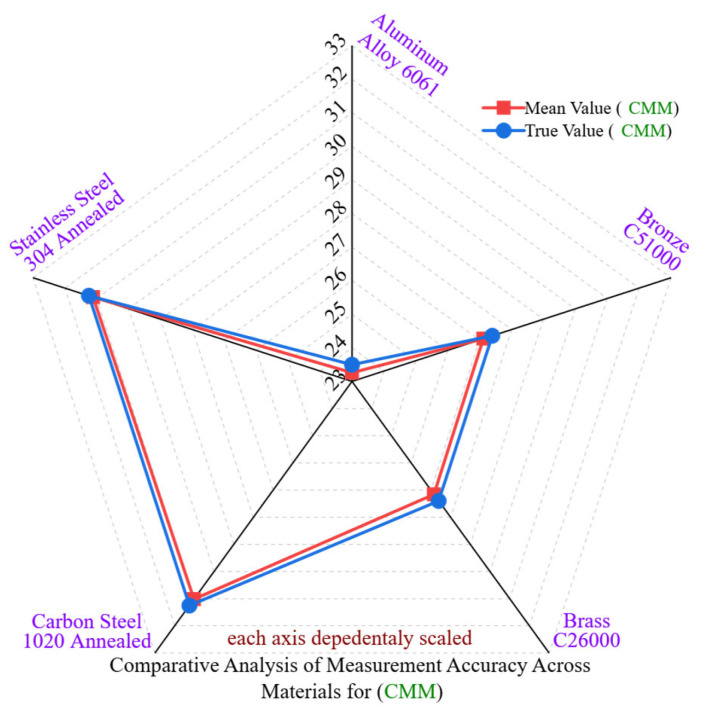
Comparative analysis of measurement accuracy across materials for CMM.

Figure 4 illustrates a radar-based comparative analysis of dimensional accuracy for the coordinate measuring machine (CMM) across five different engineering materials. In addition to absolute deviation, this analysis includes standard deviation (SD) and coefficient of variation (CV) to assess the consistency and variability of measurements.

Aluminum Alloy 6061 demonstrated the highest accuracy, with a deviation of −0.206 mm for CMM and −0.2397 mm for DVC. The standard deviation was ±0.00177 mm for CMM and ±0.02974 mm for DVC, resulting in coefficients of variation of 0.0076% and 0.1279%, respectively. The consistency in CMM is anticipated, while the greater variability in DVC indicates an increase in manual error. Although aluminum offers good machinability and a consistent surface finish, the impact of operator error is more pronounced when using manual tools.

Brass C26000 recorded deviations of −0.201 mm and −0.2398 mm for CMM and DVC, respectively. The standard deviation (SD) and coefficient of variation (CV) for CMM were ±0.00238 mm and 0.0088%, while DVC values reached ±0.02912 mm and 0.1071%, respectively. The lower CV in CMM indicates consistent automation, whereas DVC’s higher CV is due to pressure sensitivity and angularity errors during measurement.

Bronze C51000, which is harder (110 HBW), showed dimensions of −0.2495 mm (CMM) compared to −0.2824 mm (DVC). The corresponding standard deviation (SD) values were ±0.00343 mm (CMM) and ±0.03333 mm (DVC), with coefficients of variation (CVs) of 0.0126% and 0.1226%, respectively. The increase in elastic recovery, burr formation, and thermal accumulation during machining likely impacted the stability of the probe and jaw.

Carbon Steel 1020 Annealed exhibited deviations of −0.2168 mm (CMM) and −0.2327 mm (DVC). Although the absolute deviations were moderate, the standard deviation (SD) for CMM was ±0.00281 mm while for DVC it was ±0.01763 mm, with coefficients of variation (CVs) of 0.0091% and 0.0569%, respectively. The lower CV for DVC is particularly noteworthy as it may indicate uniform strain patterns, although tool wear still contributed to surface variability.

Stainless Steel 304 Annealed, noted for its hardness, recorded measurements of −0.1102 mm (CMM) and −0.1312 mm (DVC). The standard deviations were ±0.00166 mm (CMM) and ±0.02793 mm (DVC), resulting in coefficients of variation (CVs) of 0.0053% and 0.0893%. Unlike DVC, the low CV for CMM indicates its ability to mitigate localized error propagation due to strain hardening and surface deformation.

These results confirm that CMM consistently exhibits not only a lower mean deviation but also substantially improved statistical stability. CMM CV values stay below 0.013% across all materials, while DVC CV values fluctuate between 0.0569% and 0.1279%, indicating that manual measurements are more prone to random error sources. This underscores the importance of combined analysis of deviation and dispersion when choosing a measurement system, particularly in high-stakes contexts. Although DVC may seem advantageous regarding mean deviation in softer materials, its reduced repeatability diminishes trust for crucial dimensional control.

**Figure 5:**
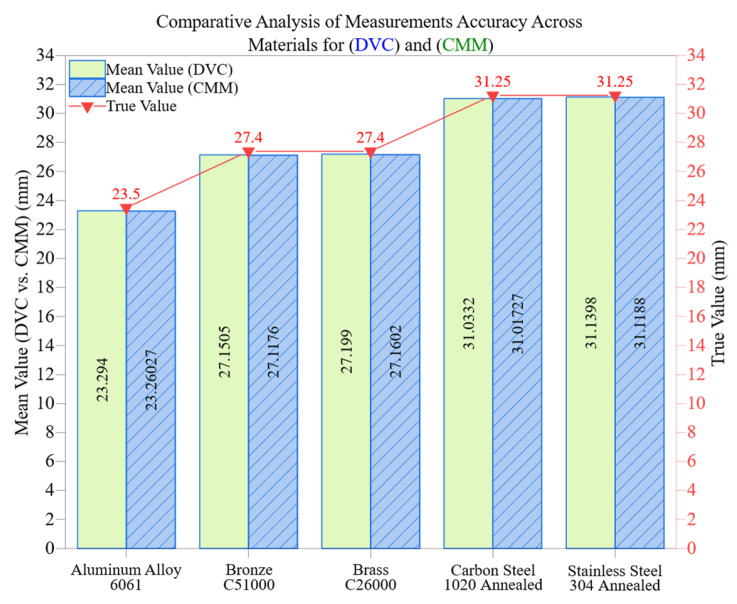
Comparative analysis of measurement accuracy across materials for DVC and CMM.

Figure 5 compares measurement deviations from the digital vernier caliper (DVC) and coordinate measuring machine (CMM) across five different engineering materials. This unified visualization allows for a straightforward evaluation of how material properties like hardness, machinability, and thermal conductivity affect dimensional accuracy and repeatability.

CMM shows consistently lower deviation and tighter clustering in all materials, indicating its high repeatability and limited sensitivity to surface anomalies. Conversely, the DVC displays wider dispersion and greater overall deviation values, highlighting its vulnerability to operator-induced variability and interaction with unstable surface characteristics.

For Aluminum Alloy 6061 the measured deviations are −0.206 mm (CMM) and −0.2397 mm (DVC), resulting in a small difference of merely 0.0337 mm. Thanks to aluminum’s high thermal conductivity of 167 W/m·K and its relatively low hardness (95 HBW), the machining process yields clean surfaces with minimal residual stress, enabling both methods to attain high accuracy. Although the DVC’s coefficient of variation (CV) at 0.1279% is higher than the CMM’s 0.0076%, it remains within acceptable limits due to the material’s consistent surface response.

Brass C26000 displayed deviations of −0.201 mm (CMM) and −0.2398 mm (DVC), creating a deviation gap of 0.0388 mm. Although brass has excellent machinability (100%) and low hardness (70 HBW) its softer surface may slightly deform under manual caliper pressure, which increases the DVC’s CV to 0.1071% in contrast to CMM’s 0.0088%. This indicates the subtle impact of tactile pressure and alignment on the accuracy of manual measurements.

Bronze C51000 is harder and more resistant to cutting, with machinability at 50% and hardness noted as 110 HBW. This material presented greater challenges for both systems. The deviations measured were −0.2495 mm for CMM and −0.2824 mm for DVC, creating a difference of 0.0329 mm. Additionally, the DVC’s CV of 0.1226% indicates significant variability attributed to burrs, work-hardened areas, and local surface curvature. Although CMM exhibited more stability overall it did report its largest deviation in this context, likely due to the probe interacting with microstructural gradients.

The difference in measurements for Carbon Steel 1020 Annealed was only 0.0159 mm (−0.2168 mm CMM compared to −0.2327 mm DVC), with the DVC exhibiting its lowest CV of 0.0569% in this case. This could be due to the uniformity of the material response and consistent cutting conditions across different zones, which helped to reduce irregularities even during manual handling.

Stainless Steel 304 Annealed, recognized for its hardness and thermal resistance, showed deviations of −0.1102 mm (CMM) and −0.1312 mm (DVC). Although it is prone to strain-hardening and poses challenges during machining, the tool difference measured only 0.0210 mm. The CMM demonstrated outstanding repeatability with a CV of 0.0053%. In contrast, the DVC’s CV of 0.0893% indicates ongoing variability due to elastic recovery and work-hardened surface layers.

These comparisons demonstrate that CMM shows much greater resilience to material-induced variability, exhibiting CVs below 0.013% across all conditions. In contrast, the DVC’s performance declines as hardness increases, thermal conductivity decreases, or work-hardening rises, with CVs nearing or exceeding 0.12%. This highlights that mean deviation alone is an inadequate measure of metrological stability, necessitating the inclusion of statistical dispersion metrics like SD and CV in performance evaluation frameworks.

Additionally, Figure 5 shows that although DVC may produce comparable mean values in certain instances, it falls short in the repeatability, traceability, and thermal/environmental compensation that modern CMM systems provide. Therefore, those choosing metrology tools should consider not just material properties but also the acceptable tolerance range, production conditions, and importance of the component’s dimensional specifications.

### 3.2. Absolute vs. Relative Error: Accuracy and Threshold Evaluation

**Figure 6:**
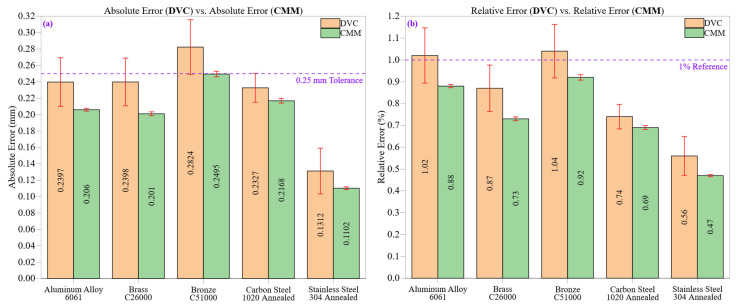
(**a**) Absolute errors for DVC and CMM across five materials; the dashed line (0.25 mm) marks the general machining tolerance per ISO 2768 [26]. (**b**) Relative errors with a 1% threshold line for standard industrial uncertainty limits.

Figure 6 offers a detailed comparison of measurement accuracy between a digital vernier caliper (DVC) and a coordinate measuring machine (CMM) for five different engineering materials. Figure 6a shows the absolute errors recorded by each system, while Figure 6b displays the corresponding relative errors. Each bar includes error bars representing ±1 standard deviation (SD), visually representing measurement variability. These subfigures collectively highlight deviations from nominal values and the statistical reliability of each measurement system.

Throughout all materials examined, the DVC usually shows slightly lower absolute error values compared to the CMM. Nonetheless, as illustrated in Figure 6a, this seeming advantage is countered by significantly greater standard deviations. For example, Aluminum Alloy 6061 reported absolute errors of 0.2397 mm (DVC) and 0.2060 mm (CMM), with respective standard deviations of 0.02974 mm and 0.00177 mm. Consequently, the DVC’s slightly lower mean error is coupled with significantly higher variation, reflecting poorer repeatability. This pattern persists in Brass C26000 and Bronze C51000 as the DVC presents higher mean errors and wider uncertainty ranges while the CMM exhibits lower average deviations and tighter statistical controls.

The higher absolute errors seen in DVC, especially in early machining zones, are largely attributed to unstable surface conditions such as burrs and tool marks which distort tactile alignment. Operator pressure variability further exacerbates this, particularly on softer materials. In contrast, CMM errors remain lower due to multipoint probing and geometric compensation, though minor deviations persist when surface waviness or anisotropy alters the probe’s contact orientation. For example, Stainless Steel 304 showed relatively small mean error but higher variability due to strain-hardened layers resisting uniform probe engagement. Similarly, Bronze C51000 exhibited error spikes related to residual stress accumulation and poor chip breakability, affecting both DVC repeatability and CMM contact stability.

The relative errors illustrated in Figure 6b further support this interpretation. For Stainless Steel 304 the relative errors were 0.56% (DVC) and 0.47% (CMM). Although the magnitude difference is slight, the variability of DVC remains higher across all zones. While DVC measurements sometimes align more closely with the nominal dimension, they lack consistency, which poses significant issues for applications needing tight tolerances or process certification. Conversely, the CMM’s reliability, bolstered by automated multipoint probing and ISO 10360-based calibration, guarantees measurement results that are both stable and traceable.

The differences in relative error (Figure 6b) follow similar patterns. DVC errors were amplified by inconsistent jaw pressure and parallax errors in visually read positions, while CMM errors correlated more with topographic irregularities. Overall, error propagation in both systems reflects a combination of material responses, machining dynamics, and system-specific sensitivities, indicating that both instrument design and surface evolution contribute to metrological accuracy.

The dashed reference line at 0.25 mm in Figure 6a indicates the machining tolerance threshold established by ISO 2768 for linear dimensions in medium classes. Materials like Bronze C51000 often approach or surpass this limit when measured with DVC, raising issues for dimensional compliance if manual tools are used in critical situations. Likewise, the 1.0% reference line in Figure 6b marks an industry-standard threshold for the acceptable relative error in a high-precision setting. Notably, DVC readings for softer materials, such as aluminum and bronze, exceed this threshold in initial machining areas, while CMM values consistently remain below it across all materials and zones.

Statistical analysis indicates that CMM standard deviations remained below 0.0035 mm across all materials, while the coefficients of variation (CV) stayed under 0.013%. In contrast, DVC standard deviations reached up to 0.03333 mm (Bronze C51000), with CVs surpassing 0.12%. These discrepancies underscore a key metrological principle: accuracy by itself does not ensure reliability. A measurement system must also reduce uncertainty and uphold consistency across different surface and material conditions. The CMM stands out in this aspect because of its diminished sensitivity to operator variation, steady contact force, and geometric compensation algorithms.

In summary, the data shown in Figure 6a,b highlight that although the DVC is beneficial for quickly inspecting ductile or thermally conductive materials, its applicability is significantly limited in scenarios that require high repeatability, traceability, and adherence to standardized tolerances. The CMM’s resilience to surface variability, residual stress, and the effects of material hardness makes it the preferred choice for dimensional verification in advanced manufacturing sectors, such as aerospace, biomedical, and mold-die industries.

**Figure 7:**
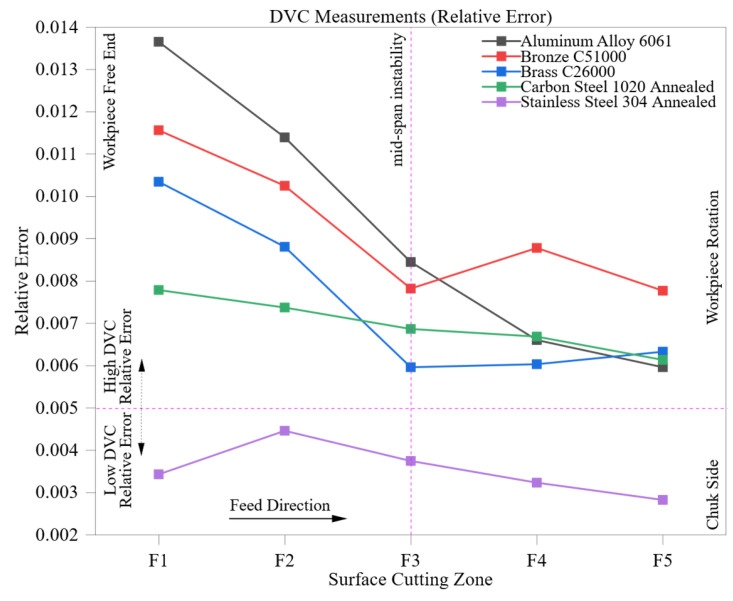
DVC measurements (relative error) across five materials and cutting zones (F1–F5).

Figure 7 depicts the changing relative error in digital vernier caliper (DVC) measurements across five CNC-machined materials and five axial cutting zones (F1 to F5). This gives a dynamic view of how manual measurement performance varies with machining progression. The identified patterns reveal the interactions among material-specific surface behavior, texture evolution due to machining, and measurement variability influenced by the operator.

For all materials the relative error shows a general decrease from zone F1 to F5, indicating that deeper machining leads to surface stabilization and improved measurement consistency. This effect is particularly noticeable in ductile, thermally conductive materials like Aluminum Alloy 6061, where the initial relative error of 0.014 at F1 drops to 0.006 at F5. The higher early-stage error is due to the unstable interaction between the cutting tool and the freshly machined surface. As machining continues, aluminum’s excellent thermal conductivity and low hardness allow for smoother material removal, lowering burrs and irregularities that could disrupt jaw alignment and tactile contact.

Similar patterns can be seen in Brass C26000 and Bronze C51000, starting with relative errors of 0.010 and 0.012, respectively, and stabilizing at 0.006 and 0.008 by F5. However, bronze demonstrates a localized spike at F4 (0.009), likely due to machining-induced burr formation, tool edge wear, or the concentration of residual stress, which temporarily affects the reliability of manual measurements. These variations highlight the vulnerability of manual techniques to localized surface anomalies, particularly in harder or strain-sensitive materials.

In comparison, Carbon Steel 1020 Annealed demonstrates a slower and more gradual decrease in error, shifting from 0.008 at F1 to 0.006 at F5. This narrower range of improvement indicates that increased hardness, cutting resistance, and related tool wear diminish the advantages gained from progressive surface refinement. Manual measurements in these materials continue to struggle due to microstructural inconsistencies and variations in surface-induced stress, which restrict measurement repeatability even with careful handling.

Stainless Steel 304 Annealed exhibits the most stable and consistently low relative error, starting at 0.0034 and slightly decreasing to 0.0028. The small deviations at F2 and F3 likely result from transient thermal fluctuations or minor tool deflections; however, stainless steel’s overall work-hardening behavior and surface uniformity enable highly consistent manual measurements. Its performance across all zones remains fully within the stability band (<0.005) shown in Figure 7, making it stand out among the tested materials as the most compatible with DVC-based assessments under controlled conditions.

These observations highlight the material-dependent characteristics of manual measurement behavior. Softer materials show greater initial variability but experience more substantial improvements with deeper machining. In contrast, harder materials provide better initial stability but offer limited progressive enhancement. The stability zone indicated in the figure delineates a practical threshold for confidence in manual metrology, assisting in determining where DVC measurements can be deemed statistically reliable.

Despite this the fundamental limitations of DVC, including jaw misalignment, variations in operator pressure, and surface sensitivity, are still significant, especially in initial machining areas or when surface defects are present. These results underscore the necessity of zone-aware measurement planning and highlight the recommendation to utilize automated systems such as CMM in high-precision settings where maintaining repeatability and consistency is crucial throughout the machining process.

**Figure 8:**
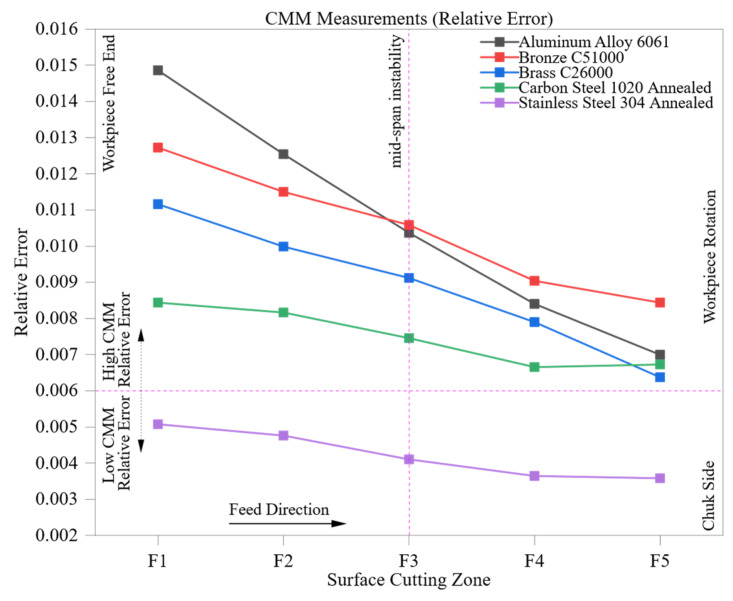
CMM measurements (relative error) across five materials and cutting zones (F1–F5).

Figure 8 illustrates the changes in the relative error for coordinate measuring machine (CMM) readings across five axial machining zones (F1 to F5) for the specified engineering materials. In contrast to manual measurement systems, CMM consistently demonstrates low relative error and high repeatability, even as surface conditions change during ongoing cutting. The findings verify the system’s ability to withstand machining-related thermal and geometric irregularities that often undermine manual metrology.

In the case of Aluminum Alloy 6061 the relative error starts at 0.015 in zone F1 and gradually decreases to 0.007 by zone F5. This enhancement indicates a reduction in surface micro-irregularities, like burrs and waviness, which are commonly generated during the initial tool engagement phase. The alloy’s high thermal conductivity and low hardness aid in achieving a uniform surface finish, while the CMM’s multipoint probing technique guarantees precise dimensional capture as the surface becomes more stable.

Brass C26000 and Bronze C51000 demonstrate similar declining trends, with initial values of 0.011 and 0.013 in F1 and decreasing to 0.006 and 0.008 by F5, respectively. Bronze experiences a brief plateau at F3 (0.011), possibly resulting from localized machining issues, such as tool wear or interactions at microstructural phase boundaries. Still, the CMM preserves dimensional accuracy due to its high-resolution scanning and automated compensation algorithms, which remain unaffected by isolated surface features that might disrupt manual measurements.

The initial relative error in Carbon Steel 1020 Annealed is 0.008, flattening to around 0.007 in the later zones. Due to this material’s increased hardness and cutting resistance, mild distortions or surface-induced stresses may occur; however, the CMM’s geometric adaptability and thermal stability help to minimize their effects. This stability additionally reinforces the use of CMM systems in medium–hard ferrous alloys, where manual tools frequently face challenges with repeatability due to varying surface conditions.

Stainless Steel 304 Annealed exhibits a consistently low relative error profile, with values from 0.005 to 0.004. While it has low thermal conductivity and is prone to work hardening, the CMM shows remarkable consistency. This indicates a successful handling of potential issues like localized hardness gradients and slight surface warping, issues which can greatly impede manual measurement systems.

The data confirms that CMM systems provide accurate and consistent measurements during the machining process independent of material hardness, thermal conductivity, or tool–material interaction effects. The relative error either consistently decreases or stays constant, demonstrating both progressive surface refinement and the system’s resistance to operator influence, tactile misalignment, or a variation in surface contact.

The persistently low relative error rates among all five materials highlight the essential function of automated metrology in high-precision applications. In industries like aerospace, biomedical device production, and defense manufacturing, where strict dimensional tolerances and traceability are crucial, CMM technology provides unparalleled reliability and consistent measurements regardless of the zone, proving vital for in-process verification, certification, and quality assurance.

### 3.3. Dispersion Metrics and Repeatability: A Statistical Assessment

**Figure 9:**
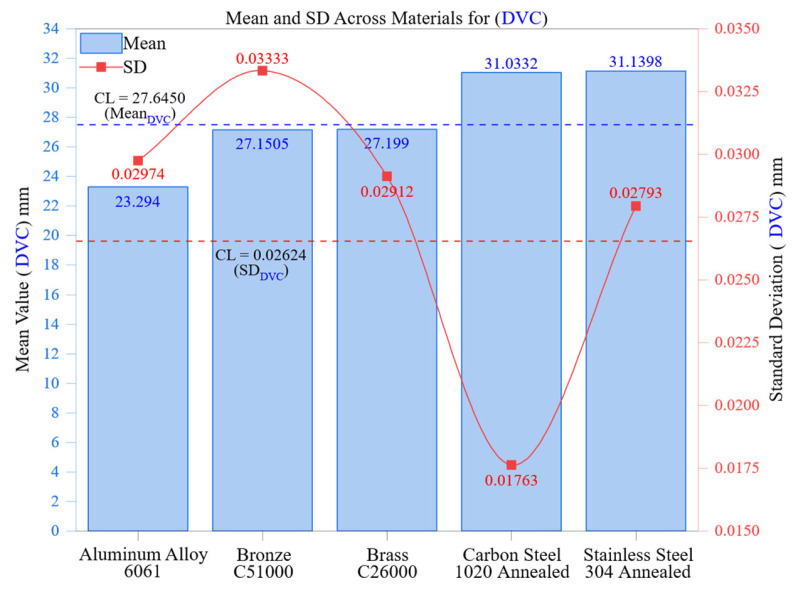
Mean and SD across materials for DVC.

Figure 9 shows the average dimensional measurements and their corresponding standard deviations (SDs) obtained with a digital vernier caliper (DVC) for five different engineering materials. This analysis illustrates the connection between material properties and the repeatability of manual measurements, utilizing SD as a statistical measure to describe the variance of repeated readings. The findings emphasize that while manual tools are simple and readily available, they are vulnerable to variability caused by the operator, especially as material hardness and machinability change.

Aluminum Alloy 6061 exhibited the lowest mean deviation, recorded at 23.294 mm, and had a standard deviation of 0.02974 mm. This relatively small variation showcases aluminum’s excellent machinability, high thermal conductivity, and low hardness, which all contribute to smoother surface creation and more reliable tactile feedback during manual probing. Despite natural variability in manual processes, the consistent surface quality and minimal burr formation in aluminum enhance repeatability, illustrating that softer materials with thermally stable surfaces can alleviate some of the challenges of manual systems.

Brass C26000 and Bronze C51000 reported average measurements of 27.199 mm and 27.1505 mm, with standard deviations of 0.02912 mm and 0.03333 mm, respectively. These moderate standard deviations indicate an intermediate level of machinability and hardness in these alloys, which exhibit greater resistance to cutting and are prone to localized surface irregularities like burrs, micro-steps, and shallow tool marks. Such irregularities hinder the consistency of DVC jaw placement and pressure application, especially during repeated measurements. Despite a mean value similar to brass, the slightly higher standard deviation noted in Bronze C51000 can be explained by its lower machinability index (50%) and greater work-hardening tendency, which increase the likelihood of operator-induced misalignment during measurement.

Carbon Steel 1020 Annealed and Stainless Steel 304 Annealed, the hardest materials in this group, showed the largest average dimensions, measuring 31.0332 mm and 31.1398 mm, respectively. Their standard deviations (SDs) were 0.01763 mm and 0.02793 mm, highlighting a complex trend. Carbon Steel exhibited the lowest SD among the group, but it is essential to consider the measurement context: steel surfaces may provide greater tactile stability due to their rigidity. Nonetheless, tool wear and residual stress accumulation contribute to variations in roughness, particularly at the free end of the machined area. Conversely, Stainless Steel 304, which is known for its work-hardening characteristics and low thermal conductivity, displays unpredictable surface reactions after machining, complicating manual measurement consistency. The increased SD in stainless steel likely indicates a mix of material stiffness and localized strain effects that affect DVC seating uniformity.

The overall control limit line (CL = 0.02624 mm) illustrated in Figure 9 serves as a benchmark for acceptable variability among all materials. Materials exhibiting SD values exceeding this line, like bronze and aluminum, indicate a higher degree of variation in repeated DVC readings, implying a tendency toward inconsistencies in operator alignment or applied pressure. In contrast, materials with SDs falling below this limit, especially Carbon Steel, demonstrate relatively stable measurement profiles. However, this interpretation should be approached with caution, taking into account the material’s resistance to cutting and variations in surface finish roughness.

This analysis confirms that standard deviation is a crucial metric for evaluating measurement reliability, especially in manual systems like the DVC where human factors are the main sources of error. While the DVC is a valuable tool for quick inspections, its ability to deliver consistent results significantly declines when handling materials with high hardness, work hardening, or surface anisotropy. Additionally, the results emphasize the preference for automated systems, such as coordinate measuring machines (CMMs), in precision applications where consistent repeatability, sub-micron resolution, and traceable accuracy are essential.

This analysis enhances our understanding of how the variability in material response affects mechanical and thermal properties, thus influencing metrological stability. These insights are crucial for selecting measurement tools and managing uncertainty budgets in quality control systems, especially when assessing dimensional tolerances in high-value manufacturing environments.

**Figure 10:**
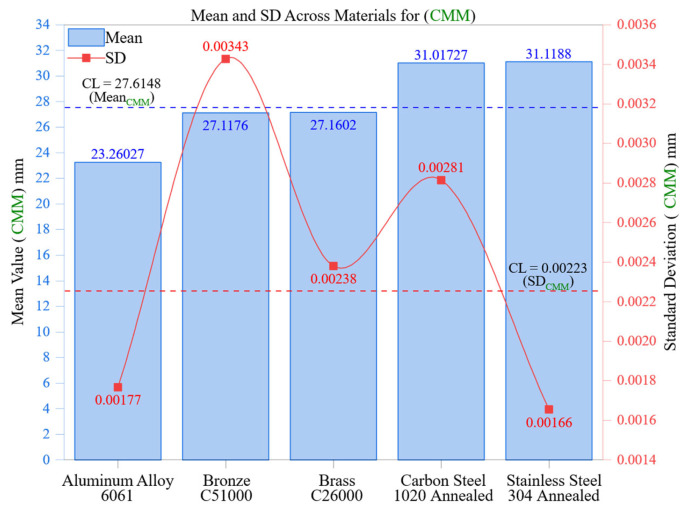
Mean and SD across materials for CMM. The bar heights represent the mean values of measured dimensions with the explicit values in dark blue, while the red line and markers denote the corresponding standard deviations (SD). Dashed lines indicate the overall control limits (CL) for the mean and SD.

Figure 10 displays the average dimensional values and their associated standard deviations (SDs) recorded with a coordinate measuring machine (CMM) across five engineering materials. This analysis reinforces automated measurement systems’ greater reliability and accuracy over manual methods, especially in managing variability across different material and machining scenarios.

Among all of the materials, Aluminum Alloy 6061 recorded the lowest mean value of 23.2603 mm, with a standard deviation of 0.00177 mm. Its properties, including low hardness (95 HBW), high thermal conductivity (167 W/m·K), and superior machinability, provide uniform surface finishes that enhance CMM probing repeatability. The notably low standard deviation indicates the capacity of CMMs to capture dimensional data with minimal deviation regardless of operator handling or localized measurement judgment, factors that typically disrupt manual readings.

Brass C26000 and Bronze C51000 showed mean values of 27.1602 mm and 27.1176 mm, with standard deviations of 0.00238 mm and 0.00343 mm, respectively. While these materials exhibit moderate machinability and mechanical stability, the somewhat higher standard deviation values, especially in bronze, may be attributed to surface imperfections such as burr edges or areas of localized tool interaction, both of which introduce minor variability. Nonetheless, these values stay well within the DVC control limits, underlining the reliable performance of CMM even with moderately challenging materials.

Carbon Steel 1020 Annealed and Stainless Steel 304 Annealed are identified as the hardest and least thermally conductive materials in this study. Their mean deviations of 31.0173 mm and 31.1188 mm correspond to standard deviations of 0.00281 mm and 0.00166 mm, respectively. Despite the presence of higher cutting forces and work-hardening effects that typically impair measurement conditions, the CMM demonstrated exceptional repeatability. Notably, Stainless Steel 304 had the lowest standard deviation among all materials tested even though it posed the greatest machining challenges. This indicates that the CMM’s multi-point scanning and surface-adaptive compensation algorithm effectively captures precise dimensional data, even in scenarios where manual tools suffer from inaccuracies due to stress and operator misalignment.

The control limit line shown in Figure 10, which indicates the average SD value for all materials (CL = 0.00223 mm), acts as a statistical standard for evaluating dispersion. Every CMM measurement remained within or below this control limit, demonstrating their remarkably low variability. In comparison to the DVC (Section 3.3), where several materials surpassed the associated CL of 0.02624 mm, the CMM revealed a tenfold drop in measurement dispersion. This illustrates that the CMM is less impacted by both material-related and operator-related variability.

Taken together, these findings demonstrate that CMM systems are not only precise in estimating mean values but also consistently stable across various materials, cutting resistances, and surface conditions. The repeatability of the system, even amidst roughness or hardness variations caused by machining, highlights its crucial role in high-precision metrology and quality control in manufacturing. CMMs mitigate key error factors associated with manual methods, such as inconsistent force application, parallax error, and subjective alignment, by utilizing fully automated, sensor-based geometric acquisition that ensures both traceability and repeatability.

The results shown in Figure 10 quantitatively support the use of CMMs in sectors demanding precise dimensional conformity, including aerospace, biomedical, and advanced mechanical systems. The CMM consistently maintains standard deviations below 0.0035 mm across materials with different machinability levels, showcasing a reliability that manual tools fail to provide, especially under conditions of compromised surface integrity or intricate geometries.

### 3.4. Integrated Performance Evaluation and Metrological Recommendations

The comparison of a digital vernier caliper (DVC) and a coordinate measuring machine (CMM) across five engineering materials showed notable differences in measurement behavior based on material properties, machining processes, and the system’s design. Although the DVC is popular due to its ease of use, cost-effectiveness, and portability, it exhibited significantly higher variability than the CMM, especially with materials that have high hardness, low thermal conductivity, and work-hardening characteristics.

CMM systems consistently show better measurement stability and repeatability with all materials, yet their practical limitations should be recognized. They come with significantly higher acquisition and maintenance costs, need skilled personnel for programming and operation, and require stable environmental conditions to ensure accuracy. These limitations may hinder their widespread adoption in small-to-medium enterprises or on shop floors lacking sufficient infrastructure. Consequently, while CMMs are optimal for critical applications that demand high dimensional precision, DVCs are still advantageous for initial checks, quick assessments, and budget-sensitive operations.

Among all of the materials tested the DVC showed greater standard deviations (SDs) and coefficients of variation (CVs), suggesting a reduced level of repeatability. While the DVC sometimes produced slightly lower absolute error values, these were frequently negated by a larger amount of measurement variability, particularly in the initial machining zones (F1-F2) where burrs, surface roughness, and gradients of residual stress were more significant. This observation underscores a fundamental metrological principle: achieving low absolute error does not guarantee high measurement quality unless it is paired with stability and repeatability.

In contrast, the CMM provided exceptional consistency and accuracy across all materials and machining zones. Standard deviations remained below 0.0035 mm, with CVs consistently under 0.013%, even for difficult alloys like Stainless Steel 304 and Carbon Steel 1020 Annealed. This outstanding stability highlights the CMM’s strength in handling machining-induced topographical irregularities, supported by volumetric accuracy, thermal compensation, and automated multi-point sampling capabilities.

The performance by zone further highlighted the differences. The relative error in DVC measurements exhibited a decreasing trend from F1 to F5, indicating surface stabilization as machining advanced. This decrease was more pronounced in ductile and easily machinable materials such as Aluminum Alloy 6061 and Brass C26000, while it was slower in harder materials like Bronze C51000 and Carbon Steel 1020 where surface recovery was hindered by greater cutting resistances and thermal effects. In comparison, the CMM showed nearly stable relative error trends, underscoring its lack of sensitivity to surface changes, even in high-hardness materials.

The findings indicate that a hybrid metrology approach is beneficial. Manual instruments like the DVC are still suitable for initial or field measurements, particularly for easily machinable materials with moderate tolerance needs. Conversely, for high-precision, valuable components, common in aerospace, biomedical, or die-and-mold applications, CMM systems are essential. They ensure traceable, low-uncertainty, and consistent results across different material conditions, which meets the requirements of Industry 4.0 quality assurance protocols.

Table 2 consolidates the essential quantitative indicators for both systems and all materials, encompassing mean measurement values, absolute errors, standard deviations, CVs, and zone-wise relative error trends. The comparative matrix emphasizes the variation in central tendency (accuracy) and the differences in statistical dispersion, highlighting the importance of assessing both aspects when choosing a measurement system for precision manufacturing.

The practical implications of these findings extend beyond traditional inspection planning. By understanding how material properties and machining zones affect measurement stability, manufacturers can design adaptive metrology workflows that optimize the use of DVC and CMM systems. These insights lay the groundwork for integration with intelligent manufacturing platforms where measurement strategies are dynamically aligned with production requirements. In Section 3.5 this approach is further developed through a multivariate PCA-based classification model, enabling predictive tool selection and more intelligent process control.

### 3.5. Advanced Statistical Modeling of Measurement Behavior

To explore the complex structural relationships between material properties and dimensional measurement results a principal component analysis (PCA) on a multivariate dataset was conducted. This dataset included absolute error, relative error, and standard deviation from both DVC and CMM systems, as well as Brinell hardness and thermal conductivity for the five materials tested. This dimensionality reduction aimed to visualize material clusters defined by similar metrological characteristics and pinpoint which variables have the greatest impact on measurement performance during CNC machining.

**Figure 11:**
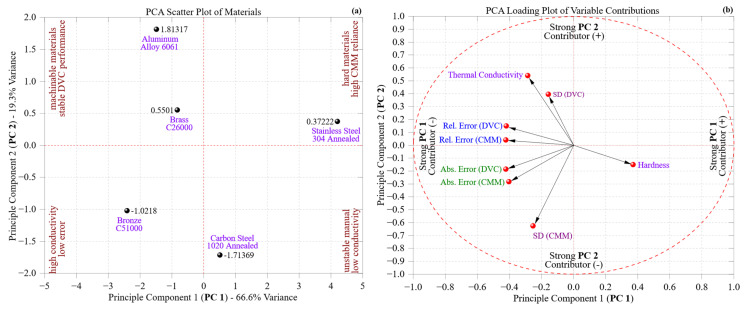
PCA of measurement and material behavior: (**a**) PCA scatter plot of the materials based on combined metrological parameters (absolute error, relative error, and standard deviation) and intrinsic properties (hardness and thermal conductivity), where purple text denotes material names, brown annotations indicate thermal/mechanical groupings and red dashed lines mark the origin of the principal components and (**b**) PCA loading plot of variables contributions to PC 1 and 2, where green labels represent absolute error, blue labels denote relative error, pink labels indicate standard deviation, purple labels correspond to material properties (e.g., thermal conductivity and hardness), and the red dashed circle outlines the correlation limit for variable contributions.

Figure 11a displays the distribution of materials within a two-dimensional principal component space. The first principal component (PC1), which explains 66.6% of the total variance, primarily differentiates materials based on manual measurements and surface sensitivity variabilities. In contrast, the second component (PC2), accounting for 19.3% of the variance, highlights differences related to automated systems’ stability and materials’ intrinsic responses. This biplot demonstrates a distinct progression from ductile and thermally conductive materials, such as Aluminum 6061 and Brass C26000, on the left side of PC1, characterized by low DVC variability and smooth surface behavior, to high-hardness alloys like Carbon Steel 1020 and Stainless Steel 304 on the right, where the consistency of measurements increasingly relies on automated probing.

Bronze C51000, situated at the center of these extremes, exhibits a hybrid metrological profile. Although it has a copper alloy lineage similar to brass, its increased hardness and machining difficulty align it more closely with ferrous alloys regarding surface-induced measurement deviations. This unique positioning captures its transitional behavior, especially in the later machining areas where burr formation and microstructural distortion affect DVC repeatability.

Figure 11b displays the PCA loading plot, which elucidates the influence of each variable on the distribution presented in Figure 11a. Vectors like SD (DVC), absolute error (DVC), and relative error (DVC) show a strong presence on PC1, indicating that manual measurement instability significantly dominates this component. These vectors extend to the edge of the unit circle, reinforcing their prominent explanatory role. In contrast, SD (CMM), hardness, and thermal conductivity align more closely with PC2, implying that this component embodies the combined effects of material hardness and the CMM’s adjustment behavior. The nearly orthogonal arrangement of DVC- and CMM-related vectors emphasizes that the two systems capture fundamentally different aspects of error propagation and measurement sensitivity.

Figure 11a,b together provide a new multivariate angle that enhances previous univariate and bivariate analyses. While earlier sections analyzed specific system performance trends, PCA demonstrates how composite measurement–property relationships categorize materials into unique behavioral zones. This structural insight directly informs the selection of intelligent metrology; materials positioned in the upper right quadrant of Figure 11a, which exhibit high hardness and CMM-dependent precision, necessitate strong, repeatable inspection protocols with limited operator influence. In contrast, materials in the lower left quadrant can be effectively measured using DVC under typical shop-floor conditions.

This modeling strategy enhances the study’s methodological contribution by merging dimensional deviation metrics with physical properties into a cohesive statistical framework. It allows for the predictive categorization of materials according to their metrological signatures, providing manufacturers with a proactive approach to synchronize measurement systems with material challenges. This predictive classification is particularly pertinent to adaptive manufacturing and Industry 4.0 frameworks, where inspection methods must flexibly adapt to the component complexity, strict tolerances, and variability resulting from processes. Essentially, PCA corroborates previous discoveries about individual system performance while enhancing the conversation by identifying latent behavioral dimensions that influence material-specific metrological results. This dual-plot framework presents a scalable, data-driven approach to refining quality control strategies in precision manufacturing settings.

To deepen the analysis of the principal component structure, Table 3 presents the percentage contribution of each original variable to PC1 and PC2, along with their physical interpretations. The first principal component (PC1), which accounts for the largest portion of variance, is overwhelmingly shaped by manual measurement parameters. Specifically, relative and absolute errors from the DVC system each contribute approximately 17–18% to PC1, confirming that operator-dependent inconsistencies, such as misalignment, pressure variation, and tactile sensitivity, are the primary drivers of measurement variability in manual inspection. Relative errors of CMM, while derived from an automated system, also contribute to PC1, suggesting that even automated measurements may be indirectly influenced by surface artifacts in poorly machinable materials.

In contrast, PC2 is primarily governed by SD (CMM) at 39.23% and thermal conductivity at 29.05%, indicating that variability in the automated system is more strongly associated with surface topography and thermal transport properties. Materials with low thermal conductivity tend to retain heat during machining, leading to increased burr formation, thermal distortion, and reduced dimensional fidelity. The standard deviation of DVC also contributes significantly to PC2 (15.60%), suggesting that surface morphology influences both systems, but more acutely in the context of automated probing. Interestingly, while contributing 13.84% to PC1, hardness has limited influence on PC2, indicating that mechanical resistance affects measurement stability primarily in manual systems, likely through its impact on tool wear and resulting surface variability.

These findings reinforce the orthogonal nature of PC1 and PC2 as the former is dominated by the operator-centric variability in manual measurement, whilst the material–property interactions that influence automated system reliability are reflected in the latter. This decomposition confirms the multivariate structure implied by PCA and provides actionable insight for metrology system selection in precision machining workflows.

Beyond visualizing the data structure, the PCA framework introduced in this study offers a scientific contribution by uncovering latent relationships between material properties, measurement error behaviors, and machining zones. This multivariate insight enables a predictive classification of materials according to their metrological stability across tools, bridging the gap between descriptive dimensional analysis and intelligent inspection planning. From an engineering perspective this approach supports data-driven decision-making in manufacturing environments by recommending optimal measurement strategies based on material response, contributing directly to the goals of adaptive quality control under Industry 4.0.

## 4. Conclusions

This research thoroughly compared digital vernier caliper (DVC) and coordinate measuring machine (CMM) systems to evaluate dimensional accuracy in CNC-turned components across five different engineering materials and various machining zones. By utilizing both absolute and relative error analysis, standard deviation, coefficient of variation (CV), and sophisticated statistical modeling, this study identified key differences in the metrological reliability, sensitivity, and performance based on zones between manual and automated measurement systems.

The CMM demonstrated remarkable stability, consistently keeping standard deviations below 0.0035 mm and CV values below 0.013%, irrespective of material hardness, thermal conductivity, or machining artifacts specific to certain zones. This dependability results from its multi-point probing feature and its resistance to surface irregularities. In contrast, DVC measurements exhibited significant variability, with SD values hitting 0.03333 mm and CVs surpassing 0.12%, especially in tougher or strain-hardened materials such as Bronze C51000 and Stainless Steel 304. Although DVC sometimes showed lower absolute errors, these were offset by increased uncertainty and diminished repeatability, particularly in the early (F1) and late (F5) machining zones.

Importantly, the application of principal component analysis (PCA) introduced a new aspect to the study by uncovering hidden connections between material characteristics and measurement behavior. PC1 represented variance associated with inconsistencies in manual measurements, whereas PC2 indicated how well automated systems compensated and how materials responded. This multivariate approach facilitated the predictive classification of materials according to their measurement profiles, providing a strategic approach for selecting intelligent metrology in high-precision manufacturing environments. The PCA further clarified these relationships by quantifying how specific variables, such as DVC repeatability, hardness, and thermal conductivity, contributed to the principal components. This not only supported visual trends in the PCA plots but also reinforced the physical interpretations needed for data-driven metrology planning. PC1 captured the variance associated with manual measurement instability, while PC2 reflected material properties and their influence on automated inspection.

In advanced manufacturing system, the PCA-derived material classifications can be embedded into intelligent inspection algorithms, allowing real-time measurement strategies to be adapted based on predicted process variability. This supports integration with manufacturing execution systems (MESs) and digital-twins, fostering closed-loop metrology planning that improves production efficiency, reduces waste, and enhances dimensional compliance, all of which are hallmarks of Industry 4.0 manufacturing.

The present work indicates that dimensional accuracy should be evaluated by its closeness to nominal values and through a comprehensive approach that incorporates statistical dispersion, material behavior, and system-specific reliability. While DVC is useful for initial evaluations within moderate tolerances, the CMM is still the favored option for critical applications that require precise traceability and process stability.

Moreover, this study advances both metrology science and engineering by illustrating the interplay between machining zones, material properties, and metrological methods, which affects the quality of dimensional measurements. The application of PCA offers a predictive strategy for selecting intelligent measurement systems. These insights facilitate better metrology choices and promote data-driven quality assurance processes in manufacturing settings aiming to adhere to Industry 4.0 principles.

## Figures and Tables

**Table 1:** Physical properties of the selected engineering materials.

Material	Thermal Conductivity (W/m·K)	Hardness (HBW)	Machinability Rating (%)
Aluminum Alloy 6061	167	95	90
Brass C26000	109	70	100
Bronze C51000	63	110	50
Carbon Steel 1020 Annealed	51	126	65
Stainless Steel 304 Annealed	16	190	45

**Table 2:** Comparative summary of measurement metrics across materials and systems. The arrow (→) in the Relative Error (F1→F5) column indicates the change in relative error from Zone 1 (F1) to Zone 5 (F5), highlighting measurement variability across different Zones.

Material	System	Mean Value (mm)	Absolute Error (mm)	SD (mm)	CV (%)	Relative Error (F1→F5)
Aluminum Alloy6061	DVC	23.2603	0.2397	0.02974	0.1279	0.0140 → 0.0060
	CMM	23.2940	0.2060	0.00177	0.0076	0.0150 → 0.0070
Brass C26000	DVC	27.1990	0.2398	0.02912	0.1071	0.0100 → 0.0060
	CMM	27.1602	0.2010	0.00238	0.0088	0.0110 → 0.0060
Bronze C51000	DVC	27.1505	0.2824	0.03333	0.1226	0.0120 → 0.0080 (spike at F4)
	CMM	27.1176	0.2495	0.00343	0.0126	0.0130 → 0.0080 (flat F3)
Carbon Steel 1020 Annealed	DVC	31.0332	0.2327	0.01763	0.0569	0.0080 → 0.0060
	CMM	31.0173	0.2168	0.00281	0.0091	0.0080 → 0.0070
Stainless Steel 304 Annealed	DVC	31.1398	0.1312	0.02793	0.0893	0.0034 → 0.0028
	CMM	31.1188	0.1102	0.00166	0.0053	0.0050 → 0.0040

**Table 3:** Percentage variable contributions to PC1 and PC2 with physical interpretations of their influence.

Variable	PC Contribution (%)		Physical Interpretation
	PC1	PC2	
Abs. Error (DVC)	17.73	3.46	Major contributor to PC1: reflects instability in manual measurement accuracy.
Abs. Error (CMM)	16.21	8.02	Impacts both PCs: CMM error sensitivity increases with complex geometries.
Rel. Error (DVC)	17.50	2.20	Strong PC1 influence: DVC relative error captures operator inconsistency.
Rel. Error (CMM)	17.80	0.16	Primarily PC1: reflects misalignment and parallax sensitivity in DVC.
SD (DVC)	2.44	15.60	Dominant on PC2: higher DVC spread in rougher or irregular zones.
SD (CMM)	6.36	39.23	PC2 driver: SD (CMM) reflects surface feature interaction in precision systems.
Hardness	13.84	2.27	PC1 influencer: hardness correlates with surface resistance and tool wear.
Thermal Conductivity	8.12	29.05	Strong PC2 influence: higher thermal conductivity promotes efficient heat dissipation during machining, leading to smoother surface formation and reduced measurement variability.

## Data Availability

The data presented in this study are available on request from the corresponding author due to privacy and legal reasons.

## Abbreviations

The following abbreviations are used in this manuscript:

CNC	Computer Numerical Control
DVC	Digital Vernier Caliper
CMM	Coordinate Measuring Machine
ISO	International Organization for Standardization
NMIs	National Metrology Institutes
E_0_, MPE	Maximum Permissible Error in Length Measurement (ISO 10360-2)
PFTU	Probing Form Test Uncertainty (ISO 10360-5)
*R*_a_	Arithmetic Average Surface Roughness
SD	Standard Deviation
CV	Coefficient of Variation
GUM	Guide to the Expression of Uncertainty in Measurement
PCA	Principal Component Analysis
MESs	Manufacturing Execution Systems
ISO 1	Standard Reference Conditions For Dimensional Metrology (20 ± 0.5 °C; 50 ± 10% RH)
ISO 230-1	Test Code For Machine Tools: Geometric Accuracy And Positioning Tests
ISO 230-3	Thermal Effects On Machine Tools: Measurement Of Thermal Drift
ISO 3650	Gauge Blocks: Calibration And Specifications For Grade 0 Accuracy
ISO 3685	Tool-Life Testing: Standard Flank Wear Criteria (e.g., VB = 0.3 mm)
ISO 10360-2	CMM Performance: Maximum Permissible Length Measurement Error (E_0_, MPE)
ISO 10360-5	CMM Probing Performance: Probing Form Error (PFTU) For Contact Probes
ISO CNMG120408-PM	ISO Designation For Tungsten Carbide Inserts (80° Rhombic, Negative Rake, TiAlN-coated)
ISO 2768	General Tolerances for Linear, Angular, and Geometric Dimensions in Machining
